# Novel pervasive scenarios for home management: the Butlers architecture

**DOI:** 10.1186/2193-1801-3-52

**Published:** 2014-01-25

**Authors:** Enrico Denti

**Affiliations:** Department of Computer Science and Engineering (DISI), Alma Mater Studiorum – Università di Bologna, Viale Risorgimento, 2, Bologna, 40136 Italy

**Keywords:** Home management, Ambient intelligence, Energy saving, Coordination infrastructures, Multi-agent systems, Pervasive computing, Gamification, Smart homes, Smart living

## Abstract

Many efforts today aim to energy saving, promoting the user’s awareness and virtuous behavior in a sustainability perspective. Our houses, appliances, energy meters and devices are becoming smarter and connected, domotics is increasing possibilities in house automation and control, and ambient intelligence and assisted living are bringing attention onto people’s needs from different viewpoints.

Our assumption is that considering these aspects together allows for novel intriguing possibilities. To this end, in this paper we combine home energy management with domotics, coordination technologies, intelligent agents, ambient intelligence, ubiquitous technologies and gamification to devise novel scenarios, where energy monitoring and management is just the basic brick of a much wider and comprehensive home management system. The aim is to control home appliances well beyond energy consumption, combining home comfort, appliance scheduling, safety constraints, etc. with dynamically-changeable users’ preferences, goals and priorities. At the same time, usability and attractiveness are seen as key success factors: so, the intriguing technologies available in most houses and smart devices are exploited to make the system configuration and use simpler, entertaining and attractive for users. These aspects are also integrated with ubiquitous and pervasive technologies, geo-localization, social networks and communities to provide enhanced functionalities and support smarter application scenarios, hereby further strengthening technology acceptation and diffusion.

Accordingly, we first analyse the system requirements and define a reference multi-layer architectural model – the Butlers architecture – that specifies seven layers of functionalities, correlating the requirements, the corresponding technologies and the consequent value-added for users in each layer. Then, we outline a set of notable scenarios of increasing functionalities and complexity, discuss the structure of the corresponding system patterns in terms of the proposed architecture, and make this concrete by presenting some comprehensive interaction examples as comic strip stories. Next, we discuss the implementation requirements and how they can be met with the available technologies, discuss a possible architecture, refine it in the concrete case of the TuCSoN coordination technology, present a subsystem prototype and discuss its properties in the Butlers perspective.

## Introduction

In most of today’s systems and applications, energy management basically means energy saving: the aim is to measure how much energy is consumed, either by a single appliance or the whole house, to make the user aware and promote virtuous behaviours. In fact, several solutions exist that enable users to monitor their home energy consumption in real time – from low-cost, pass-through plugs that embed a meter to monitor a single appliance, to systems exploiting an inductive sensor in the mains line to monitor the whole house consumption via Wi-Fi (Efergy 2012), supporting the download of data to the user computer for further analysis, as well as the Web-based and app-based access to such data – including their social sharing. Other solutions, like (Scottish Power 2012), (Enel 2012) and (PG&E 2013), enable remote access to real-time energy consumption from the Web, often supporting smartphone and tablets for alerts or energy consumption tracking, sometimes exploiting for the purpose the intelligent electricity meters installed in some countries^a^.

Some research platforms, like Powerpedia (Weiss et al. 2012), integrate the energy monitoring aspect with community sharing, so that users can compare their consumption with others and possibly share them with a user community, making raw data more easily understandable and significant thanks to mutual comparison. Other systems support user-defined policies, which take into account the rate plan, the user habits, and sometimes priorities among home appliances to perform appliance control: in (Futursoft 2012), for instance, the real-time monitoring of appliances’ consumption is used to control their switch-on/switch-off times, disabling pre-determined appliances to run the most energy-demanding tasks at the cheaper times. Still, despite their effectiveness, these systems usually embed little or none intelligence – they just react to selected situations by applying pre-determined rules. Some systems, like (EyeOn 2012), go a little further, coupling load control to pre-established priorities to switch appliances on/off based on user-defined rules: yet, any change requires a system reconfiguration, since priorities cannot be changed dynamically.

The starting point of this paper is that there is much more about energy management, and home management in general, than energy saving. One first aspect is that in several countries home energy meters also enforce a power threshold, forcing disconnection if the consumed power exceeds the contract limit^b^: this might cause several problems, from computer data loss if disconnection occurs unexpectedly, to frozen food rotting if the disconnection occurs when nobody is at home and persists for some hours, to more critical situations if the blackout occurs at night (difficulties in reaching the control panel, often positioned at the building basement, to reset the meter) or involves children (risk of fear or unexpected behaviour in the dark) or elderly people (risk of falling, running up against something, etc.). To face these aspects, energy monitoring is a necessary brick, since no prevention can be made without awareness (Enel 2012) (Energy@Home 2012); but basing prevention only on the users’ awareness of a potentially dangerous situation is clearly insufficient, as recognised by some domotic systems (e.g. (Tecdomotica 2012)) that allow the controlled disconnection of appliances to prevent overloads and blackouts. Although some systems also support priorities, the list of appliances to be disconnected in case of need is mostly pre-determined, or they are selected from a pre-determined list. Further approaches, are more focused on Web-based control and tele-diagnostics, enabling users to access and control each appliance remotely, again including alarms if pre-determined critical situations occur.

The second aspect is the forthcoming development of connected, and possibly smart, appliances, aimed at exchanging real-time information with the user and/or the “house” – and sometimes also outside, typically in the context of smart appliances and smart houses (see below): the purpose is to improve awareness and possibly allow feedback actions, including forms of remote appliance control (Efergy 2012) – for instance, air conditioning systems controllable via a smartphone app, etc.

The third pillar consists of home control and automation systems, whose goal is to control home appliances in some way – not necessarily having energy saving as the main purpose: in fact, in most cases their goal is to enhance the human comfort, either automating some user actions (Ché et al. 2010) (Molesini et al. 2010) and repetitive tasks or improving human/appliance interaction, aiming at transforming the house into a smart environment (Menon et al. 2012) (Molesini et al. 2010) (Enel 2012).

In the smart home/smart cities horizon, this area is getting increasing attention by the major industries: in recent consumer electronics shows, like IFA 2013, intelligent home appliances were presented that communicate to provide advanced behaviour and comfort features for a smarter lifestyle (LG Newsroom 2013), possibly exploiting cloud services as the support for innovative services, like an “intelligent personal concierge that takes care of small matters” (NewsPanasonic.Net 2013). In the same perspective, many smart entities are being proposed in similar contexts – from smart fridges that can track their content and possibly place the order at the supermarket, to smart doors and windows, and many others.

From a different, but related, perspective, Ambient Assisted Living (Ayala et al. 2012) considers home automation and smart living as means to support elderly people, helping them maintain their independence, while pervasive home security (Carchiolo et al. 2010) focuses on surveillance systems for home (and corporate) security. Mobility and geo-localisation, now available practically on any end user device, are also generating new pervasive applications every day, providing at little/no cost advanced services that would have been simply unconceivable otherwise. Such applications are often integrated with social networks, sometimes with humanized avatars, and tend to generate their own social network to re-shape their user virtual world and experience. This opportunity is becoming well evident to the industry, as demonstrated both by the general trend towards the development of attractive apps to access services, rather than accessing them via a standard – possibly mobile – Web browser, and by some commercial services derived directly by entertainment apps: an example is the T-Net service (T-Net Drive 2013), originated by an app created for entertainment purposes (Artie World 2013).

Our view is that there would be an extra value in considering all these aspects together, putting them in context with other key research results from intelligent agents, multi-agent systems, coordination technologies, on the one side, and “entertainment” aspects, on the other – the so-called *gamification*^c^, intended as “applying game design thinking to non-game applications to make them more fun and engaging” (Gamification 2012), which is more and more making the difference between success and failure in technology acceptance and broad diffusion from the consumers’ viewpoint.

In this novel scenario, a *home management system* should enable users to perform all the above tasks in an *integrated*, *highly configurable*, easy-to-use, *pervasive* fashion. Users should be able to specify their preferences *according to their own goals and priorities* – from energy saving to protection, from comfort-oriented choices regarding heating or air conditioning to practical aspects such as having the washing machine finished when they arrive at home, so that they can switch on the air conditioning or the oven to get a pizza with their friends without violating the electricity contract limit, etc. They should also be able to do so *in a natural way*, interacting with the system easily – either in their natural language, possibly exploiting the voice recognition facilities available in any smartphone, or writing short messages like SMSs or tweets in a Twitter-like *#tag* fashion, or maybe writing longer phrases in English, Italian, French, etc. – and possibly exploiting some advanced forms of gesture-based interaction provided by todays’ gaming consoles, 3D screens, etc. Moreover, users should be able to do so *dynamically*, free to change their mind and programs at any time, because our everyday life is often unpredictable and any of us can be late, forget something, or decide to watch a TV match with friends in ten minutes, and so on. Even more, it should be possible to do this *in mobility*, without having to go home to reconfigure the system or change an option, because most of us spend far from home most of the day – and smartphones are already in everyone’s pocket. Since smartphones embed geo-localisation and are also “the” entry point for all social networks, their availability opens further intriguing possibilities – from geo-related home management services, to the *social sharing* of user experiences, in several senses.

The envisioned system should thus be seen as an intelligent “home director”, a sort of *butler* that exploits its knowledge of user preferences, priorities, habits, and user’s current location – via geo-localization or grabbing his location info from Foursquare, Google Places, and other social networks – both to enforce the specified policies and to *anticipate the user’s decisions* for his comfort (e.g. “he’s coming home earlier, let us switch on the heating/air conditioning”), safety (“no hot items”, “prevent any blackout”, etc.) or any other goal. In fact, if a suitable inferential engine is provided, this system could also anticipate or propose decisions based on the user’s current behaviour (e.g. “he’s just bought a take away pizza, he might like to find a hot oven when he arrives: let us ask him”). Generally speaking, it should do so by *coordinating* and *governing* the home appliances according to the user’s dynamically-changeable goals – including energy saving as a special case. In this view, users should no longer be forced to specify details such as whether to turn off/on the washing machine rather than the air conditioner: they should just set the desired goal (e.g. “have the wash done by 6 p.m. when I come home, with a 24°C home temperature”), and disregard the rest, letting the system take care of the details. At the same time, users should remain in touch with their butler in both directions – to be informed of any problem/situation and, conversely, to inform it if they change their mind/plans – via smartphones, social networks, etc.

Accordingly, in this paper we present the *Butlers architecture* – an architecture for a pervasive home management system defined coherently with the above perspective. The architecture is intentionally devised to be general and technology-neutral so as to account for many possible systems – each with its own policies and objectives, based on the vendor’s trade-offs between cost, performance, reliability, robustness, etc. For this reason, special attention will be paid to the available technologies that could be used for the purpose.

Moreover, recognising that technology owes much of its success to people’s acceptance and enthusiastic adoption, and that the broad diffusion of a technology among consumers is strongly related to its ability in being perceived as “entertaining” and “pleasant” other than just useful and effective, we intentionally consider the gamification aspect as one further, essential requirement. This is why our scenarios include, for instance, humanized appliances with avatars and customizable personalities, which can be personalised from the style and language viewpoints and shared in social networks so as to involve users into a 360° experience; for the same reason, the system configuration and the user interaction can take advantage of the presence of 3D screens, gaming consoles, etc.

## State of the art and related work

Monitoring devices for house energy consumption are available from several vendors: typically, they exploit an inductive sensor in the mains line, and transmit data at the least to a remote metering unit via Wi-Fi, but possibly also to some Web-based service or mobile app like in (Efergy 2012). Some electricity providers offer their own specific hardware and software apps to remotely access real-time energy consumption also in mobility, sometimes with alarm support (Scottish Power 2012). In countries where intelligent electricity meters have been or are being installed, electricity providers may offer solutions that exploit this infrastructure (Enel 2012) (PG&E 2013): understandably, efforts are being made (Meters and More 2012) to promote an open communication protocol for interoperable intelligent meters which is being considered as a potential standard by the Open Meter European project (Open Meter 2011). While the main goal is typically to monitor energy consumption, some approaches support advanced forms of tele-diagnostics and control – though more from the installer’s viewpoint than from the end user’s viewpoint – and/or the remote control of single appliances, like in the case of (Scottish Power 2012), both for energy saving and remote controlling purposes. More recently, the remote control of single appliances is being added to metering units, too (Efergy 2012).

In the Energy@Home consortium (Energy@Home 2012), energy providers, telecommunication providers and appliance vendors developed a platform for consumption control and overload prevention. Preventing overloads and blackouts is also among the goals of domotic systems like (Tecdomotica 2012), which support the controlled disconnection of appliances – in some cases, including static priorities, in others, like (Tecdomotica 2012), controlling UPS units and emergency lights, as well as light control based on environmental conditions. Energy saving and cost control are also among the main objectives of (Futursoft 2012), which operates by controlling the on/off times of “isles” of appliances based on the user configuration. The idea of constantly controlling the energy absorption is further developed in (EyeOn 2012), which considers appliances’ priorities in deciding the loads to exclude and re-activate to prevent blackouts.

From another perspective, research on Multi-Agent Systems (MAS) also considered energy balancing and control as an interesting application field, though possibly more from the energy provider’s viewpoint than the final consumer’s. In (Tolbert et al. 2001), a scalable MAS is presented for the control of distributed energy resources to achieve higher reliability, power quality, and more efficient power generation and consumption; in (Nagata et al. 2000), a multi-agent system is proposed for power system restoration after faults. The MAS approach is also used for load management in power grid systems (Zhang et al. 2011) to combine local intelligence with coordination (Papadopoulos and Arbab 1998) (Omicini and Papadopoulos 2001) (Busi et al. 2001) issues, to integrate the advantages of centralized and decentralized architectures so as to achieve accurate decisions and quick response while avoiding a single point of failure. The same goal, from a different perspective, can be found in (Li et al. 2008), while in (Biabani et al. 2012) the aim is to accommodate distributed generation resources to reduce the peak power demand.

In (Conte and Scardozzi 2007), authors investigate the use of a MAS to characterise the notion of home automation system, exploiting cognitive agents to react to user commands and explicit alarm situations. Notably, this seems to be the only work where respecting a power threshold is considered a fundamental goal (quoting Conte and Scardozzi: *“Operation of different appliances must be organized and scheduled in such a way to keep the global electric load within the limits established by the supplier, to avoid possible shut-down”*), including priorities to capture the user policies (*“the washing machine should not be allowed to get hot water if this may cause a sudden, unpleasant reduction of the water temperature the while the user is taking a shower”*).

Domotic and home automation systems also aim to automate the user interactions with home appliances. In (Ché et al. 2010), focus is on detecting (and learning) patterns in the user interactions, exploiting prediction algorithms to predict and possibly automate the next action; in (Menon et al. 2012), the goal is to identify and track the home occupants via unobtrusive recognition modalities such as face, gait, and voice, enriched with spatio-temporal reasoning techniques. In (Carchiolo et al. 2010), instead, focus is more on home and corporate security, since the goal is to design a pervasive surveillance system. Learning human behaviour patterns is also one of the main goals of (Bien and Lee 2010), which focuses explicitly on the contribution that smart home systems can bring in facilitating the independent living of the aged population. In fact, Ambient Assisted Living is a typical applications area requiring context-aware, open, scalable, and distributed software technologies incorporating intelligent and autonomic reconfiguration techniques: in (Ayala et al. 2012), a self-configuring agent system is proposed for tracking elderly people’s activity using common commercially available electronic devices.

Widening the focus towards smart environments and sensor-driven applications, works can be found that focus on cooperative and adaptable applications (Bartolini et al. 2012), promoting the interoperability and mutual understanding of the smart devices and spaces by exploiting the semantic relations between objects in the environment to overcome the device and space heterogeneity. Others, like (Rawi and Al-Anbuky 2012), focus on wireless home automation networks, i.e. the kind of networks where embedded wireless sensors and actuators are coupled together to monitor and control the home living environment, mostly to maintain the user comfort and achieve home efficiency. In (Rawi and Al-Anbuky 2012), in particular, the purpose is to inject intelligence – in the form of a fuzzy-rule-based system – to measure the human (thermal, visual, indoor air, acoustical) comfort indices.

## Requirements

In this Section we extract from the above goals the requirements of the envisioned home management system, focusing on six major areas: the general architectural requirements, the coordination requirements, the configuration requirements, the GUI requirements, the main functional requirements, and the gamification requirements. Later in this paper (Section Implementation), we will discuss whether and to which extent they can be met with the available technologies.

### General architectural requirements

From the architectural viewpoint, the basic requirements can be summarised as follows:

1. each appliance should be equipped with a consumption sensor;
2. sensors should be able to communicate with some sort of central hub, and possibly also among themselves, via some kind of communication link; this requirement is independent from the actual technology and protocol selected for the purpose, which may be chosen in a wide variety of available technologies (for instance: ZigBee, Wi-Fi, Ethernet on PowerLine Homeplug/ Homeplug AV, etc.);
3. a coordinator should take care of gathering information from such sources, embedding and enforcing the laws that govern the system and guarantee the respect of user preferences and policies. (Alternatively, approaches based on *stigmergy* (Holland et al. 1999) (Grassé 1959) – a form of self-organization that produces apparently intelligent behaviours without explicit planning or control, exploiting the environment as an indirect coordination medium – could be considered, but investigating this option is outside the scope of this paper: interested readers can find some extra detail below).

#### The stigmergy approach in a nutshell

Stigmergy (Grassé 1959) is a form of self-organization that produces complex, apparently intelligent behaviours without the explicit intervention of any planning, control, or direct communication, exploiting the environment as an indirect coordination medium. The basic idea is that each action performed in an environment affects (leaves traces in) the environment itself, possibly triggering other actions by the same or other agents: such action chaining leads to the emergence of coherent, apparently systematic activities and efficient collaboration among (very simple) agents that have no intelligence or individual awareness. The most famous application is probably the ant colony case (Dorigo and Stützle 2004), but others interesting examples range from group work (Elliott 2006) to a recent mix with a chemistry-inspired approach for coordination purposes (Mariani and Omicini 2013).

### Coordination requirements

Given the central role of the coordinator, its requirements involve several aspects – from architectural and interoperability issues, to malleability and inspectability of policies, security, up to proper support to intelligence. More precisely:

1. the coordinator should be designed in such a way to prevent the risk of creating bottlenecks – that is, it should be logically “central”, but possibly distributed at the implementation level if appropriate;
2. the coordinator should be based on open communication protocols, so as to be interoperable with potentially any appliance from any vendor;
3. the coordinator should be accessible and easily configurable by the (non-expert) user both locally and remotely;
4. the coordinator should support dynamically-modifiable, user-definable policies, inspectable and readable by any user in a clear human-understandable format, so that policies may be easily reviewed, integrated, and replaced at any time both locally and remotely; so, the coordinator architecture should not embed any hardwired technology nor any pre-installed “locked configurations”, in favour of a flexible, open configuration support; (by the way, this requirement would fit very well with the adoption of declarative specifications, possibly handled by some kind of “intelligent core”);
5. in addition, the coordinator should be able to inter-operate and cooperate with other users’ coordinators installed in other houses to exchange best practices, users policies and other relevant information – provided that an adequate security model is set up and the proper permissions are granted (security in modern houses is a hot research topic, see (Denning et al. 2013) for an in-depth discussion); such a cooperation should be started both on a user-triggered basis, as well as, potentially, as a result of an autonomous decision of the coordinator itself: to this end, integration with communities and social networks is highly desirable, both as a means to exchange data, and as a source of raw information for further reasoning;
6. the coordinator should also be able to take full advantage of portable devices’ geo-localization features to support advanced reasoning functions – for instance, deducting from the user’s positions and movement that he/she is coming home earlier, thus autonomously activating the heating/air conditioning system, re-scheduling the other activities as appropriate, etc.; (this requirement includes the ability to inter-operate with apps and social networks based on geo-localization, such as Foursquare or Google Places, for the same purpose, discussed below);
7. the coordinator could also be “intelligent” – that is, able to reason on the current situation (environment, user’s location, etc.) based on the user’s priorities, goals, but also on the current communication and coordination state (Papadopoulos and Arbab 1998) (Omicini and Papadopoulos 2001): this is the typical playground of multi-agent technologies and infrastructures, where agent autonomy and coordination tools are effectively put together (Ciancarini et al. 1999);
8. finally, in order to support the implementation of the advanced services outlined in the previous section, the coordinator should be able to interact with the main social networks (Twitter, Foursquare, Facebook, Google+, etc.) either directly or, more likely, via some ad hoc “proxies” – agents, in the MAS terminology and perspective.

### Configuration requirements

The configuration process involves several aspects – namely, simplicity, proper exploitation of available devices, local vs. remote access, up to language aspects. More precisely:

1. the configuration process should be simple, guided by a wizard if appropriate, and carefully designed so as not to be boring or annoyingly long; it should also take explicit advantage of any modern device (smartphones, tablets, but also Smart TVs, gaming consoles, etc.) that users might possess;
2. the local configuration process, in particular, should be able to exploit the Smart TVs, gaming consoles and personal computers available in the house, as well as other more pervasive devices like smartphone and tablet (via suitable apps or ad hoc Web sites), as well as via a voice menu system able to guide the user towards the desired configuration in a natural and familiar way; presence sensors could also be exploited to detect the user’s position in the house and activate the interaction device(s) available in that or the closest room;
3. the remote configuration process, on the other hand, while exploiting the same smartphones & tablet apps and the Web sites cited above, could take advantage of other specific means, like shorts text messages (SMS – available in virtually any cell phone including the oldest ones), instant messaging systems, “twitter like” messages (with proper tags to be defined), as well as the user’s natural language thanks to the smartphones’ built-in text-to-speech/speech-to-text facility;
4. the configuration language – intended as the set of commands usable to control the configuration process – should be linguistically expressive and efficient, that is, flexible enough to support a wide variety of different appliances and policies, enabling any useful command and situation to be expressed while retaining ease of use and overall simplicity; in addition, it should also be clearly uncoupled from the user’s natural language, both to easily support any users’ nationality – even simultaneously – and to preserve the underlying layer from changes in the users’ natural languages.

### Graphical User Interface requirements

In order to fulfil the above requirements and support the envisioned scenarios, the GUI should take several, integrated forms:

1. physically, it should support both local – exploiting available appliances such as Smart TVs (possibly 3D sets), wall screens, touch screens, gaming consoles, voice systems – and remote access – via text messages, short messages, voice messages, apps, wizards, Web sites, etc.;
2. conceptually, it should make it easy and natural for the user to express high-level goals, desires and preferences (e.g. “make sure that the washing machine finishes by this evening, not later than 7 pm”), in addition to specific detailed commands (like “switch this device on”, “turn the radio off”) or command sequences (e.g. “do this before that”), promoting high-level specifications that delegate the coordinator for (most of) the practical scheduling: this is not only desirable from the user’s viewpoint, to free the user from having to plan detail aspects (most likely, in an un-optimized or sum-optimal way), but also to provide the coordinator with the necessary degrees of freedom;
3. it should also enable the simultaneous specification of multiple goals – for instance, “try to do this, but at the same time save as much energy as you can”; or “do these things together, but don’t cause blackouts”, etc. – yet in a clear, easy-to-learn and understandable way – that is, no room for complex multi-dialog interfaces or that kind of multi-level menus that are often found in DVD recorders, video-cameras, and many other consumer electronics, that most people never use (if they know where they are..) because they are “too complex to understand”.

### Functional requirements

Although several functional requirements have already been mentioned indirectly, in terms of desired functionalities and system features, it is worth summarising them here to provide a complete view:

1. the system (model and controller) counterpart of the GUI should support the expression of high-level goals, desires and preferences – rather/other than detailed commands and command sequences – from the linguistic viewpoint, including priorities among home appliances, user-defined constraints, etc.: accordingly, an expressive and easy-to-use language should be available, that is naturally usable from several different interface platforms – from text based messages to Twitter-like messages, touch screens, voice systems, up to posts on Facebook or similar social networks;
2. dynamic system reconfigurations (user goals, policies, priorities, etc.) should be supported at any time, and be possible through a variety of media, locally and remotely, both as a result of a user will or of an autonomous system decision;
3. the specification of multiple simultaneous goals should be supported, in pair with a conflict detection and resolution mechanism that takes into account the user goals, policies, priorities, and constraints;
4. geographic information must be properly handled and exploited – that is, grabbing the user location from either explicit position information or via indirect indications like posts about commercial activities, Foursquare check-ins, etc.; if possible, this information should also be stored in such a form that it can be easily and efficiently exploited as a base for behaviour detection, to infer the user’s behaviour (e.g. “he is coming home”) provided that an intelligent inferential engine – possibly an agent in a MAS perspective, or maybe the coordinator itself – is included in the architecture;
5. optionally, an intelligent inferential engine – if present – could support advanced features such as anticipating the user’s desires, propose actions to achieve the user’s goals beyond the mere application of pre-compiled plans, help reducing conflicts, etc.; as mentioned above, this engine might conceptually take rather different forms at the architectural level, ranging from some ad hoc component (a sort of “expert system” or an agent with special capabilities) to a distributed system where intelligence is spread in the coordination architecture, up to the “extreme” case of the stigmergy approach.

### Gamification requirements

Last, but not certainly least, an adequate support to the *gamification* of the user experience, including the entertainment aspect under several forms, should be included. As discussed above, this choice aims to achieve several pervasive-oriented objectives:

1. to broaden the social sensibility to the energy saving & management theme in general, and among the younger generations in particular, promoting the users’ interaction with the main social networks and communities;
2. to promote the interaction with the community(-ies) of users, to exchange suggestions, best practices, etc.;
3. to enhance people’s acceptance and voluntary technology adoption, by making the user experience more attracting, appealing and involving, ‘’humanizing” interactions.

#### Appliance communities

To this end, appliances could be “humanized” and equipped with a personality – which could be as complex and involving as many character traits as desired, possibly inspired to the user’s own personality – and “put in the arena” both to support multi-user gaming on some suitable platforms, and to play on social networks in a post-based, Facebook-like fashion. Note that participating to *today’s* communities could be just the starting point of some new kind of “mixed” (human/appliance) social network, which could evolve in forms hard to guess today. In principle, appliance communities could grow up with different goals and forms:

- ***level 0***: merely technical communities, aimed at exchanging practical information (policies, practices) with no expected human interaction (possibly except for some technical operator, from time to time): just a little more than a conventional communication protocol, no particular humanization required;
- ***level 1***: mixed appliances/human communities, where humanized appliances – somehow similar to comic/cartoon strip characters – interact together, still with no particular autonomy or intelligence: this is where personalities, avatar and gamification could start to play a role;
- ***level 2***: enhanced version of the above communities, with humanized appliances expressing also some form of autonomy or intelligent behaviour: personalities could then evolve “on their own”, beyond being just the pure projection of the home user/appliance manufacturer.

#### Appliance humanisation

In its turn, appliances humanisation could occur in very different, yet worth exploring, directions – which could take inspiration for their style and traits from comic, movie and cartoon characters, both in terms of icon, personality, and communication style. In particular, we could have ironic, verbose, or logic (“Vulcanian”-style) avatars, which communicate, interact and behave as the user perceives that appliance to be: for instance, a user could see his house inhabited by a verbose washing machine, as annoying as some never-stop-talking mother-in-law, and by an English gentleman-style refrigerator, while his best friend’s house could be very different, reflecting his personal view and style.

Appliance personalities should be highly configurable in a wide variety of aspects – communication style, voice for voice synthesis systems (like today’s navigators), responsive style (eager, lazy..), mood, etc. – and virtually any desirable trait. Traits could also be moody, based on their expected “usage happiness”: for instance, an oven could very happy when expecting to be used to cook a pizza for a nice dinner with friends, and be sadder when unused for some time; and so on. Such aspects could also evolve over time to follow the user’s mood – which is actually what people already do every morning when posting their feelings and day life facts on Facebook, Google+, etc. – as well as their bigger life changes, like moving to a new house, or relevant success/failures in work or personal life which influence their life perception and happiness: for instance, the appliance personalities of the house of a single man/woman could change notably if he/she finds a partner and starts a new life, etc.

As a next step, appliances personalities could be exported and *shared* among users in the social networks and in the user communities, becoming themselves a means to strengthen the communities, reinforce the entertainment effect (leading users to feel protagonist) as well as the users’ proactivity; as a side effect, humanized appliances embedding policies or preferences could become a way to promote and support policy/preferences sharing, with a multiplier effect on pervasivity. One further way to improve pervasivity and thrill/involve users could be to support on-line games and tournaments among humanized appliances, seen as users’ representatives, in specific (possibly energy-oriented) games – for instance, “who saved more energy today” – possibly aimed at showing off abilities – e.g. “today I rescheduled washing, oven and air conditioning eight times, to make my master happy”, etc. Clearly, in this perspective, imagination is the only limit: virtually any game and situation can be adapted to serve the purpose.

## Reference architecture

**Figure 1:**
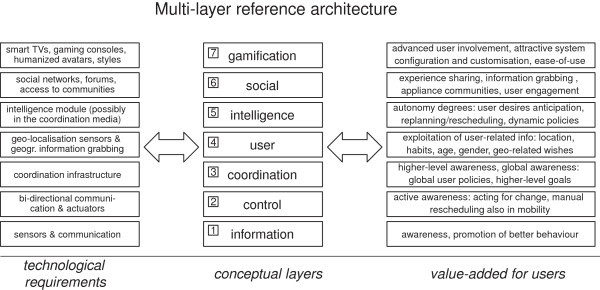
**The conceptual layers of our reference architecture and their relationships with the technological requirements (on the left) and the value-added for users (on the right).**

The resulting multi-layer reference architecture is depicted in Figure 1, where the conceptual system layers (at the center) are related both to the technological requirements (on the left) and to the consequent valued-added expected for the user (on the right). System realisations are not required to imperatively cover all layers: in fact, any actual system is free to choose how far to go, possibly leaving empty levels in specific cases, based on the application’s own goals and priorities.

The bottom layer (*information*) is the enabling one: indeed, most of the systems reviewed in Sections Introduction and State of the art and related work operate at this level, allowing users to check their real-time energy consumption from a multitude of media (from displays to mobile apps), and promoting energy-saving behaviours via users’ awareness.

The second layer (*control*) adds some form of remote appliance control: as discussed in the Sections Introduction and State of the art and related work, this is the typical goal of home automation and assisted living, but is also a pre-requisite for any active approach to home management that aims to go beyond the mere awareness of what’s going on in the house.

The upper layers, instead, are more independent from each other, and therefore more freely selectable. The third layer (*coordination*) is rarely considered today, except for some research systems (see Section State of the art and related work); the same holds for the fourth layer (*user*), too, which is aimed at considering “who and where” the user is. Traces of features from the fifth layer (*intelligence*) can sometimes be found, albeit in limited forms like detecting and learning user interaction patterns; analogously, traces of features from the sixth layer (*social*) can be recognised in the social sharing features (like posting and sharing the energy consumption to promote the best practices and awareness via mutual comparison of data) provided by some systems. To the best of our knowledge, the top layer (*gamification*) is mostly unconsidered today in this application field.

Although these layers are not strictly hierarchical, some dependencies among them do exist: for instance, while the basic layer is indispensable, some features from layers 5 and 6 can be present even when some of the lower layers, like 3 and 4, are empty (or nearly empty), as in some of the above-discussed cases.

## Notable scenarios and system patterns

Moving from the state-of-the-art presented above, several scenarios, of increasing complexity and pervasivity, can be imagined, which correspond to different selections of layers, and possibly different selections of the features providing the value-added for users within each single layer.

For the sake of concreteness, we discuss here some of the most intriguing possibilities, outlining a hierarchy of systems – from the basic informer up to the most comprehensive system. To express their architectures compactly, we use sum-like expressions (e.g. *A + B* + …) to indicate the required layers: more precisely, *N* represents the need for support of layer *N*, while N* indicates that only a partial support of the features inspired by level *N* is actually required.

### The informer (a.k.a. the awareness promoter)

This is barely what most of the systems surveyed in the previous Sections provide today, and is reported here just for completeness: their architectures involve layer 1 only.

### The remote controller

The basic version of this kind of system makes use of layers 1 and 2, both to be informed and to control home appliances *directly* – for energy saving, protection, comfort, or any other purpose: some of the existing systems discussed above (especially in the home automation context) do this, to some extent. However, the scenario we first considered in the Introduction goes farther, assuming that users can control their appliances *in a natural way* – that is, communicating with the system easily, in their natural language, via voice recognition, short messages, maybe phrases in English, Italian, French, etc., in mobility and dynamically. This kind of system makes still use of layers 1 and 2, but invests on the communication aspect to improve pervasivity and provide further value-added for users. As a slight enhancement, communication might include tweets or posts on social networks, thus embracing layer 6, though without actually exploiting the social aspect in depth. As a further step, avatars could be added in the appliance control GUI (both locally and remotely), moving towards the direction of layer 7, albeit without stressing this aspect deeply. Possible systems architectures in this category therefore include 1 + 2, 1 + 2 + 6*, 1 + 2 + 7*, and 1 + 2 + 6* + 7*.

### The basic butler

The second scenario adds coordination as a means to enable users to specify preferences and priorities, both statically and dynamically. This makes it possible to go beyond the mere “direct user control” of an appliance in favour of an approach where higher-level goals can be defined (like “setting the home temperature according to the user’s preferences, while having the washing machine done by 7 pm, but guaranteeing that the power threshold is always respected”) that delegate the coordination infrastructure for the consequent detailed decisions. It should be noted that the expression “coordination infrastructure” does not necessarily refer to a complete coordination infrastructure: in principle, a central coordinator, with proper communication protocols, could do as well, albeit in a less scalable and maintainable way. However, adopting an effective coordination infrastructure makes it possible to enforce the specified policies, actually governing interaction and increasing the system robustness and security. Systems in this category include the same above, with a “+3” specification in their layer description.

### The user-aware butler

In this scenario, the extra value-added comes from *knowing the user* – mainly his position, but also his age, gender, preferences, habits – and taking this information into account for deciding the activities’ coordination, so as to provide a more “user tailored” behaviour. The user location can be either obtained via geo-localisation of an enabled device, or grabbed from elsewhere – for instance, from the user’s check-ins on Foursquare or Google Places, though this choice implies some features from layer 6 – or simply obtained directly from the user, either asking or because the user provides his position proactively (even sending it by a low-level mechanism as a properly-formatted old-style SMS^d^). Personal user information such as age and gender can already be available if the user is known, or be grabbed as above for guests.

The likely effect of this information is to influence the priorities of tasks or the scheduling among appliances, favouring the ones that are most needed to meet the user comfort if he/she is coming home, and – conversely – perform the most annoying tasks when he or she is at work or at school. Examples range from controlling the home temperature to the preferred level when a given user is coming home, to warming the oven if the chef lady is coming home and likes to cook a cake or a pizza, scheduling the washing machine when nobody’s at home provided that there’s enough time for it before dinner time, etc. However, no specific intelligence – intended as the ability to reason and anticipate possible user wishes – is considered to be present at this stage: decisions are driven only by the rules embedded in the coordination layer during the configuration stage. Thus, systems in this category are substantially the same as above, with a “+4” layer in their architecture description.

### The intelligent butler

By adding intelligence as a fundamental feature, this scenario adds the ability to anticipate the user’s decisions and wishes, exploiting any available information, taken and re-elaborated from virtually any source. This puts greater emphasis onto the specification of the user’s goals*,* which can be now of a higher level than above, and include reasoning on the current system state, on the previous actions, and, if available, on the user position, identity, and behaviour. Consequently, systems in this category would particularly benefit from geo-localisation information provided by layer 4, although this is not indispensable to provide value-added for users. Examples of such intelligent decisions can range from comfort-oriented decisions (like “his car shows that he’s coming home earlier than usual, let us switch on the heating/air conditioning”), to the concretisation of abstract safety policies into specific actions (e.g. “no hot items”, “prevent blackouts”, etc.), to deductions from the user’s current behaviour that can lead to proactively propose further actions (e.g. “he’s just bought a take away pizza, he might like to find a hot oven when he arrives: let us ask him”), and so on.

Since many advanced features depend on the user’s possibility of remaining constantly in touch with his/her butler (intended here as the user’s *alter ego,* to some extent), both to be informed of any problem/situation and, conversely, to inform it if of any change of mind, an adequate communication and interaction support is crucial. While the communication media and interaction forms outlined above for the basic remote controller can suffice, an enhanced communication/interaction support could greatly help to express and manipulate the user’s policies, preferences, desires, goals, etc. This is why intelligence could also cover the linguistic aspect, supporting higher-level concepts and metaphors: in fact, this is also essential to capture the configuration process at an adequate abstraction level, posing the base for its *“*gamified*”* support at layer 7.

Moreover, intelligence is crucial also to face information heterogeneity, bridging between different sub-system viewpoints and to support the integration of “legacy” sub-systems into a whole: this is the case, for instance, of the interaction with the social networks, considered at layer 6, as well as in other uses like the enabling of inter-coordinator interactions, etc.

So, possible system architectures in this category include 1 + 2 + 3 + 5 (minimal version), and 1 + 2 + 3 + 4 + 5 (better version), both with the possible further extension to layers 6 and/or 7.

### The social butler (and the appliance’s communities)

At layer 6, the key aspect is the integration with the social networks, in all the forms discussed above. Such integration can occur in both directions, that is, on the one hand users and appliances can participate in social activities, producing information, while on the other they can exploit communities to grab information – be it geographical information, user habits’ information, best practices, etc.

Any social network and community kind can potentially be considered in this context, from old-style forums to specific user communities, up to Facebook, Google+, Twitter, Foursquare, Google Places, etc. Adding the social dimension has a value *per se,* acting as a multiplier of the user engagement, other than as an extended source of information and user behaviour; yet, it is undeniably the major brick for building systems according to the gamification perspective, too.

The social contribution can range from basic situations (supported even in the basic remote controller scenario, and actually found in some of the existing systems) where *users* share their experience to advanced situations where *humanized entities* – maybe the single appliances, more likely the butler as the leader/representative of its community, possibly both – participate to their own communities and to traditional human communities, interacting with themselves and with the other users autonomously, within the boundaries of the pre-established security policies (in principle, policy negotiation could also be included, but that aspect is outside the scope of this paper). Such system architectures have the same form as above, with a “+6” addition.

### The comprehensive butler

Layer 7 considers the ability to entertain and make things nice as a fundamental aspect to involve people and to make complex tasks within their range. As discussed above, the main means to achieve these results are:

- user involvement, making him/her perceive to be part of the system by showing him/her that the system is perceiving him/her presence and possibly also that it knows his/her identity, habits, preferences: even a low cost sensor of presence used to activate the interaction devices in the room (TV sets, screens, etc.) can make the difference, because fun is based on engagement and on the feeling that your system is interacting precisely with you; another way could be using a custom (user-selected) voice in a text-to-speech interaction system; and so on.
- humanization of home appliances with highly-customizable avatars, whose personality, attitude traits, communication style, language, voice, etc. can be defined by the user (possibly starting from a set of pre-defined models to enable a one-minute quick start), so that they reflect his/her way of life, habits and mood;
- concretization of appliances and more abstract concepts (tasks, goals, preferences, rules, configuration aspects, etc.) into physical, (possibly 3D) entities, to be manipulated very much like characters and objects in games on gaming consoles, possibly exploiting wearable movement sensors, so as to turn complex tasks (especially the configuration of priorities, rules, etc.) into physical experiences: if opportune, scores, classifications, awards, winners and similar accoutrements could also be included. This allows for a wide variety of systems, inspired to different gaming approaches and user engagement’s philosophies; the game metaphors and level could also be tailored to the user’s age, experience, role, permissions, etc., so as to provide each user with the kind of experience that is most suited for him/her – e.g. an intriguing game for a child or a young adult, or better a reassuring quiet location for the less experienced, possibly elderly people (for instance, the grandmother’s kitchen virtual ambient), and so on;
- sharing what’s happening in social networks, under a plurality of forms – from the user personally posting what he/she is doing with his/her house system, to humanized butlers interacting with the user’s wall/diary as if it were an old friend (with their personality – see above), to appliance communities sharing best practices and policies (and why not, maybe even also curiosities..), to mixed appliance/human communities, up to humanized appliances as friends on Facebook, Google+, etc.

System architectures in this category can vary from those providing a basic support, as in the remote controller scenario (1 + 2 + 7*, 1 + 2 + 6* + 7*), to full-fledged systems involving all layers – the same above with a “+7” addition – where the gamification philosophy impacts nearly all the aspects of the system, from local interaction to remote interaction, system configuration, coordination control and reasoning rules, goals specification, experience sharing.

## Examples

In this Section we illustrate some possible “everyday interactions” with a comprehensive system like the one devised above in the form of comic strips.

### Paolo & Archie

**Figure 2:**
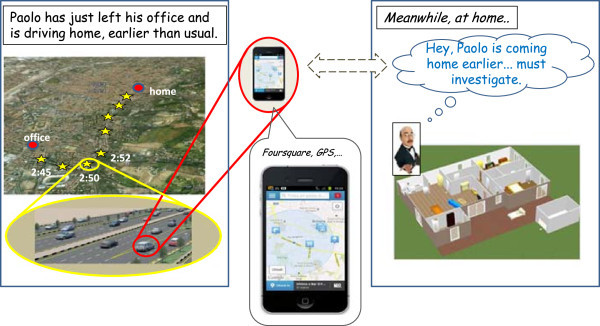
**Paolo’s butler, Archie, detects that Paolo is coming home earlier, makes a tailored guess about the possible reasons, verifies the guess via personal messaging, and orchestrates the house accordingly.**

**Figure 3:**
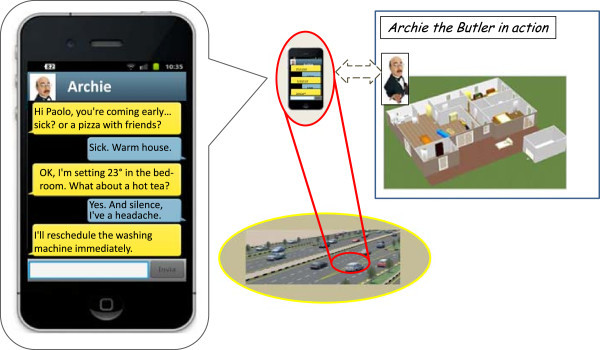
**Archie verifies his guess via personal messaging, and orchestrates the house accordingly.**

In this first example, described in Figures 2 and 3, Paolo decides to leave his office in Bologna earlier than usual (Figure 2), because he feels sick and has a terrible headache. His butler, Archie, detects this fact by tracking Paolo’s location (via GPS or some geo-localisation sites), notices that it is quite unusual and makes a guess on the reasons thanks to his knowledge on Paolo’s personality and habits. Since Paolo is a rather systematic person, who enters/leaves his office at the same time every day, Archie’s best bet is that Paolo might be sick, so (Figure 3) it prompts Paolo via some personal messaging to check its hypothesis.

Once confirmed about Paolo’s conditions and desires, the butler orchestrates the house appliances accordingly, transforming Paolo’s goals – such as “silence” in the house because of the headache – into the consequent detailed decisions – such as switching off the washing machine to ensure silence, and activate the boiler to provide a hot tea.

### Ann & John

**Figure 4:**
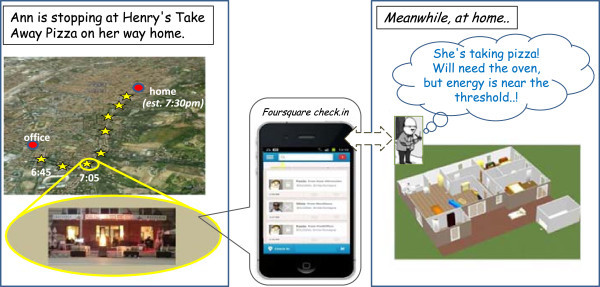
**Ann’s butler, John detects that she is buying a take-away pizza, and deducts that she will like a hot oven.**

**Figure 5:**
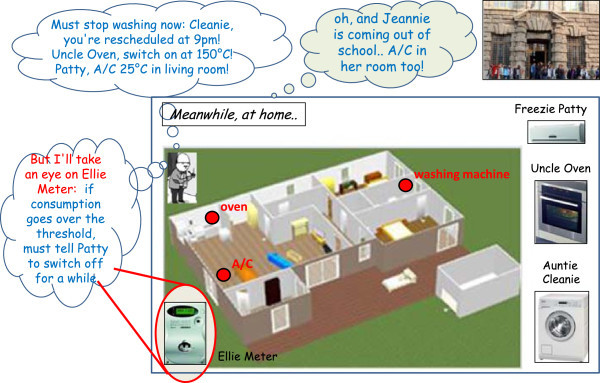
**John autonomously reschedules the home tasks, delaying the washing to allow energy for the oven; meanwhile he also keeps an eye on Ann’s daughter, Jeannie, anticipating her needs and applying the appropriate comfort policies.**

The second situation is similar to the above, but occurs in a different context. Ann is coming home normally, but decides to stop at a take-away pizza (Figure 4): her butler, John, discovers this fact by checking her check-ins on Foursquare/Google Places, or by comparing her position with its own knowledge of the city area. Hence he deducts that she will probably like to find the oven properly hot, so he switches it on, dynamically rescheduling the home tasks (Figure 5) to allow for the extra electricity consumption. To this end, the washing machine, previously scheduled at this time, is delayed so that the oven power does not cause any blackout due to the electricity threshold.

**Figure 6:**
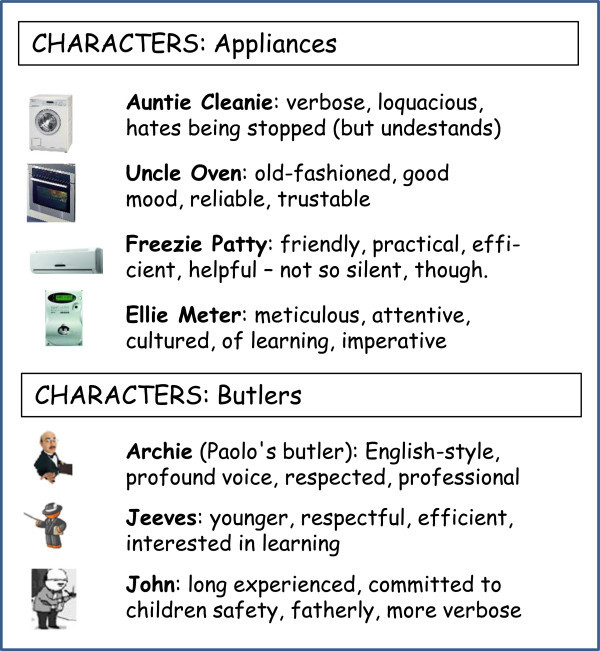
**In the gamification perspective, humanised appliances feature a precise, user-defined personality and have unique characteristics; butlers are even more humanized.**

In the gamification perspective, appliances are humanized with a proper avatar – possibly with a funny name, and feature a precise, user-defined personality (possibly including style, a specific voice, etc.), according to the legend shown in Figure 6.

Meanwhile, the butler keeps also an eye on Ann’s daughter, Jeannie (Figure 5), noticing her exit from school and applying the related comfort policies in the house so as to anticipate her needs.

### ButlersBook

**Figure 7:**
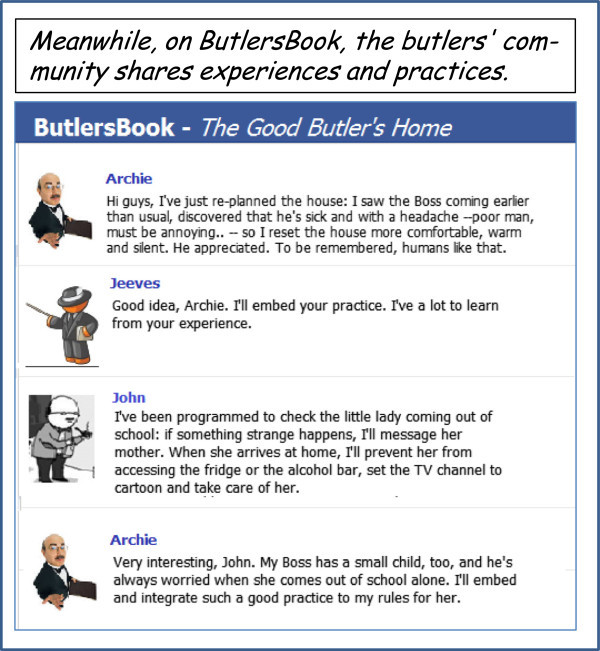
**Butlers interact to exchange experiences and best practices on** ***ButlersBook*** **, one of the butlers’ community.** Since this community is open to users, avatars are used: for the users’ fun (gamification), butlers are even more humanized than appliances.

Figure 7 shows an example of a butlers’ community, here called *ButlersBook*, where butlers interact to exchange experiences and best practices. While closed communities (as discussed above) might be used, this community is intentionally open to users, so butlers are represented as avatars, feature a rather precise personality for the users’ fun (gamification), and behave according to their own style – see Figure 6 for details on their personality and style.

Despite the fun aspect, the community actively supports the sharing and exchange of butlers’ experiences and best practices, based on their willingness to share and learn, as expressed by their personality. Appliances communities (not shown) could also be considered. Of course, all such the community are accessible via different devices – from smartphones to tablets, Web, etc.

## Implementation

As a reference multi-layer architecture, one of the main values of Butlers is its independency from any specific technology, which would otherwise limit its generality: in fact, as widely discussed above (Section Notable scenarios and system patterns), many different systems could be considered, each covering different layers and possibly realized with quite different technologies, depending on the designer’s goals and on the vendor’s market areas. On the other hand, concreteness is necessary to prove the feasibility of the Butlers’ approach, especially as concerns the integration of so many heterogeneous technologies from so many different areas.

For this reason, in the following we conjugate these two needs by presenting one possible design of a Butlers system, yet delaying the commit to a specific model and technology as much as possible, so as to keep the conceptual and the practical aspects as separated as possible. To this end, we first analyse the available technologies needed for the Butlers layers from different areas, present a possible logical architecture of a Butlers system, then commit to a specific coordination model and infrastructure – the key choice that fixes the design and interaction metaphors, and therefore the basic ingredients of the system structure. Nevertheless, the Butlers system at this stage is still “general” in the meaning of Section Notable scenarios and system patterns, in that its features can still be scaled in a range – even if its overall design philosophy and “design language” is by now fixed by the key choice above. In the next step, we consider a subset of such a system, that is small enough to be implemented “by hand” without requiring a large team effort while retaining all the essential aspects of the full system. Finally, we present the actual implementation of such a system, discussing its intended application scenario, its agent-based architecture, and outline the resulting prototype.

So, this Section is organised as follows. In Subsection Technologies we survey the main available technologies in the relevant fields, and evaluate whether – and to which extent – they can be used for our purposes. Then (in the Subsection Logical architecture of a possible Butlers platform) we present the logical architecture of a possible Butlers system implementation, discussing the relationship between the properties of the architectural components and the features that the system can consequently be expected to provide. As a next step, we commit to a specific coordination technology and infrastructure (TuCSoN) as the technology of choice to concretise our Butlers logical architecture: we summarise its basics and discuss how its main metaphors – *tuple centres* – can be exploited to effectively provide the kind of flexible and extensible support needed by our architecture. The consequent TuCSoN-based implementation is discussed in Section TuCSoN for Butlers. Then, to be even more concrete, we take a small, but significant, sub-system and present its prototype implementation. Finally, we discuss the conceptual roadmap towards a real Butlers system: starting from the prototype, we discuss how, and to which extent, its limitations could be overcome with respect to all the relevant aspects – namely, home appliances, users categories, policies, configuration issues, and the butler itself.

### Technologies

Our goal here is discussing to which extent the scenarios depicted in the previous Sections can be implemented with the available technologies, taking into account the requirements outlined in Section Requirements.

#### Sensors and communication technologies

As mentioned in the previous Sections, sensors for energy consumption measuring and monitoring are available from many vendors: so, it is reasonable to assume that each appliance can be equipped with proper sensors. The extra requirement that each appliance is provided with some kind of communication link is equally feasible, with a variety of protocols and system to choose from: since appliances are power-supplied, vendors could find it convenient to exploit technologies like Ethernet on PowerLine/Homeplug to make communication-enabled appliances with no extra wiring, but other cost-effective and widely available alternatives like Wi-Fi (or ZigBee) could be equally adopted – especially for battery-operated or gas-powered appliances.

#### Configuration technologies

Wizard technologies have long been available on personal computers, so meeting requirement #1 in Section Requirements – which is also a GUI concern – is basically a matter of good design and proper implementation: of course, a smartphone-based or tablet-based version of this wizard should be tailored to the characteristics of such devices, like touch screens. Smart TV sets (requirement #2) could be treated analogously, but the 3D technologies that are becoming more and more common both on TVs and gaming consoles could be exploited to provide more involving, innovative configuration experiences: configuration could take place by inviting users to interact with 3D entities – maybe humanized animals or appliance avatars – instead of merely navigating through classic, tedious dialogs or on-screen display instruction. The wizard could also exploit the natural language, exploiting voice recognition and text-to-speech systems to talk to the user, making the system more accessible to visually-impaired or elderly people in the Assisted Living perspective. Presence sensors are also a low-cost, widely available technology, used typically in light switching and alarm systems: yet, nothing prevents it from being exploited in a novel scenario.

Similar considerations hold for the remote configuration aspect (requirement #3), since the communication means considered in addition to the previous ones (SMS, instant messages, tweets, etc.) are everyday, low-cost technologies.

In short, all the technologies needed to face these issues are clearly available – often just in the smartphone in our pocket – so all is needed is to put everything together.

The last requirement #4 is more technical, but the required knowledge and technology are also available: domain-specific languages are an established technology, so – more generally speaking – designing an expressive and efficient configuration language is well in our reach. Keeping it separate from the users’ natural language is merely a matter of good design, and presents no conceptual difficulties.

#### User Interaction technologies

Apart from the configuration-related aspects, the above technologies can be used for other aspects of the user interface (requirement #1), provided that a consistent view is given across the various interaction media. Because of the emphasis on high-level goals, desires and preferences (requirements #2 and #3), voice could well be the preferred mean over classical menus or on-screen dialogs that most users tend to find complex and unnatural: this is particularly relevant when the intended users include elderly people, children, and other non-expert categories – quite a common situation in a house, indeed.

In addition, a “gamification-oriented” approach could also be explored – albeit with extra difficulties due to the higher abstraction level of these interactions with respect to the configuration above: in principle, goals and preferences could be concretised as items in a virtual (possibly 3D) world, so that their selection and specification becomes very much like an intriguing (strategy, but maybe also combat..) game; the kind of the game could be system-specific, or even user-specific – for instance, preferring simple games for children, and more complex 3D worlds for younger adults. From the technology viewpoint, everything we need is already there: avatars are widely adopted in many contexts, from online forums to mode complex applications, gaming consoles (often with on-board Wi-Fi connectivity) are in many houses, and so are Smart TV and 3D TV sets, wearable movement sensors that enable natural yet advanced user interaction are also spreading around, etc. So, exploiting such technologies for our purposes is mainly a matter of integration – possibly a non-trivial issue from the industrial viewpoint, but hopefully not-so-critical from the conceptual viewpoint.

#### Gamification technologies

The technologies required to properly support the gamification approach are mostly the same discussed above for user interaction: so, their availability and presence in everyone’s house have already been discussed. Appliance communities – both in the form of merely technical and mixed appliance/human communities – constitute to some extent a novel idea, but the required technology is again well established – the novelty is in integrating this aspect in the new scenario. The avatar’s customisation in terms of style, traits, personality, communication style, etc. is not particularly original *per se*, either – so, the related technology is there to be reused – but is used here in a novel application context. In particular, extending such humanisation/customisation to the communication and interaction aspects of appliances’ avatars, including physical aspects like voice, morphological details, etc. and possibly modelling their behaviour after the user’s mood, opens interesting perspectives in a social-aware scenario. Still, this does not require new technologies to be developed on purpose, but just to put together technologies available for different purposes in separate worlds.

#### Coordination technologies

Although coordination technologies are expectedly less common in consumer electronics, the topic of coordination has long been studied (Papadopoulos and Arbab 1998) (Omicini and Papadopoulos 2001) (Busi et al. 2001) (Omicini et al. 2004) in terms of models, languages, infrastructures and tools – sometimes under the *orchestration* keyword. Depending on the context and on the adopted model, the responsibility of performing the coordination activities can be either on the entities to be coordinated (also called the *coordinables*), or on some ad hoc entity/-ies (the *coordinator(s)*, whatever form they take and whatever protocols they use), or even on the *coordination media*, or a mix of these.

The first option (often referred to as the *subjective* approach), where coordination is mostly up to the coordinables, emphasizes the entities’ autonomy and demands little or no requirements from the underlying communication system (and model): it can also be easily coded in virtually any language and platform, since coordination in this case is just one more task an entity must perform. However, the overall system correctness depends on the entities’ honest behaviour and willingness to cooperate, since there is no way to force the respect of any coordination law or protocol: so, the system robustness is weak, and security issues can arise. In the opposite scenario (referred to as the *objective* approach), entities do not interact directly, but through *mediators* – at least in the initial phase: then, direct communication might occur, for efficiency reasons – that control and govern interaction, enforcing the respect of the coordination laws: this is the basic difference between *enabling* and *governing* interaction (Omicini et al. 2004). When coordination is intended in this sense, the coordination media often take on themselves most of the coordination task, unburdening the coordinables from the direct of the coordination protocols; so, these become lighter and less error-prone. One further advantage is that the coordination laws are no longer spread among the coordinables – that is, hardwired in their code, making debugging a nightmare – but can be stored in and enforced by the coordination media. This choice makes it also possible, at least in principle, to change the coordination policies by need – even dynamically, at run time – with no impact on the coordinated entities, while supporting heterogeneous agents. Last but not least, since any interaction occurs through these media, the *interaction state* can be made available and inspectable at any time for monitoring and debugging purposes – an essential condition for any coordination infrastructure to be actually usable, together with a reasonably efficient implementation.

Generally speaking, objective coordination is the typical approach of choice for complex interactive systems that have to deal with openness and heterogeneity, since the uncertainty about the number and nature of agents makes mediators nearly indispensable; the subjective approach, instead, is more frequently preferred in closed-world scenarios, where the coordinables are known a-priori, and their interaction can therefore be better analysed and engineered also without an intermediate mediator.

Given the Butler’s characteristics and application scenarios, the objective approach seems more adequate. For this reason, in the following of this paper we will adopt one such technology – the *TuCSoN coordination infrastructure* (Omicini et al. 2004) – as the conceptual and implementation support for our study of feasibility and prototype.

Of course, other alternatives could be considered (e.g. JADE (Bellifemine et al. 1999, Bellifemine et al. 2001, JADE 2013), MARS (Zambonelli 2002), TOTA (Mamei and Zambonelli 2009), CArtAgO (Ricci et al. 2009), simpA (Ricci et al. 2011) and simpA-WS (Ricci and Denti 2007), – to cite just some), but our aim here is just to guarantee that an adequate technology for building the home management system exists and can be effectively used for that purpose, not to survey and compare coordination infrastructures in general – interested readers can find such a comparison in (Omicini et al. 2004) and (Omicini 2013).

#### Other technologies

Other technologies exploited in the Butler’s scenario range from linguistic technologies to geo-localisation, information grabbing, geo-mapping technologies, up to artificial intelligence.

The linguistic emphasis in requirement #1 can exploit a wide range of technologies that are clearly available in any recent device, tablet or smartphone – from speech recognition, to text-to-speech synthesis, writing helpers, etc.; the same holds for geo-localisation capabilities (requirement #4), that are also embedded in today’s devices.

Rather, grabbing such information indirectly, by analysing the user’s posts on Facebook, Foursquare and other social networks, requires a specific technology to be integrated. While similar technologies exist – for instance, to extract information from search engine’s queries: we all see “targeted” commercial ads being proposed right after a Google search, to say one – they are not necessarily tailored to geographical information or to the user’s position: what they do is trying to infer the user’s desires to propose vacations, services, goods, etc. Other, more geographical-oriented technologies do exist, however: some adopt a similar technique to propose restaurants, hotels and tourist services after a post on Foursquare or Google Places, while others infer the user’s position via his/her current IP address: so, the above requirement can conceptually be met by integrating such technologies in a new architecture, exploiting them in the new context.

The intelligence aspects (requirements #4 and #5), despite their relevant role in the envisioned architecture, are less critical from the technological viewpoint at this stage, since decades of research in the AI, expert systems, machine learning and MAS fields have led to a strong knowledge in the area and to a wide range of models, languages, architectures, and technologies: so, designing and building a “suitable” inferential engine, with the required properties, is basically a matter of choosing the preferred approach and technology – where “preferred” can refer to many aspects (cost, platform, availability, support for rapid prototyping, open-source, etc., to cite just a few), to be valued and weighted according to the designer’s viewpoint and vendor’s marketing goals.

### Logical architecture of a possible Butlers platform

**Figure 8:**
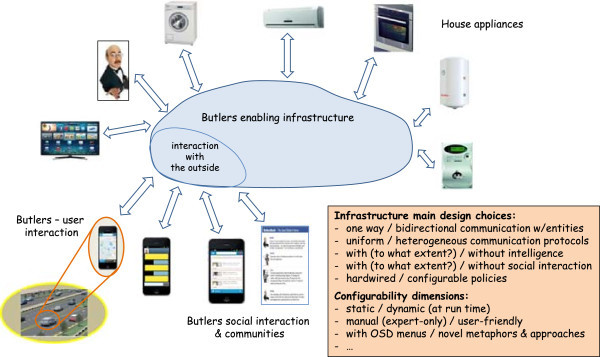
**Logical architecture of a Butlers platform.**

Following the previously-highlighted requirements and keeping an eye onto the available technologies, the logical architecture of a Butlers platform can be devised as in Figure 8. Basically, the Butlers infrastructure is aimed at enabling and governing the communication and interaction between the house appliances and their butler, on the one side, and the external world, on the other. Different design choices are possible at this stage, leading to different infrastructures and systems that cover a different set of layers in the Butlers multi-layer reference architecture (Figure 1). Some of the main design dimensions include:

- whether the communication between the entities (appliances, but also monitoring tools, apps, etc.) and the infrastructure is one way or bi-directional, for all or just some of the entities (see layers 1 and 2 of the reference architecture); in a chain, this also affects the choice whether one single protocol is supposed to be adopted, or different heterogeneous protocols are to be supported – including the addition of new protocol support to an existing system;
- whether the infrastructure to be built is basically hardwired, or, conversely, requires configurable policies (see layer 3 of the reference architecture); in the latter case, the subsequent choices concern *when*, *how* and *by whom* these policies are to be configured – in particular, whether the configuration is supposed to be performed statically or also dynamically (at run time), and whether it is up to the system administrator only or should be available also to the final users, with which rights, by which means, and with which metaphors (this also affects layer 7);
- whether some form of intelligence is to be included in the system, for which purpose, in which parts and to which extent – from the simpler form of exploiting user information to higher-level tasks (layers 4 and 5 of the reference architecture)
- whether some form of social interaction has to be considered, in which forms and for which goals (layer 6 of the reference architecture)

Different answers to these questions lead to different Butlers systems, with different features and capabilities, ranging basically over the whole set of possibilities discussed above – and beyond: any vendor can “carve” its system according to its needs and industrial aims.

**Figure 9:**
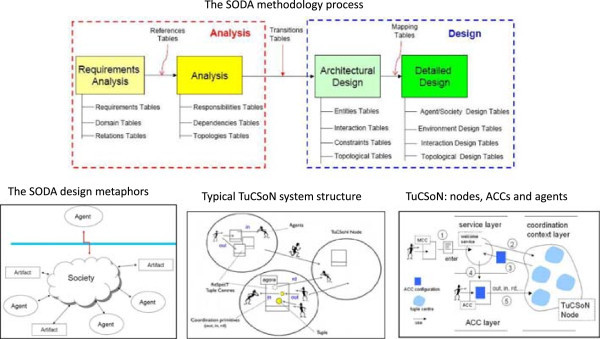
**Overview of the SODA methodology process and of the TuCSoN model and infrastructure.**

**Figure 10:**
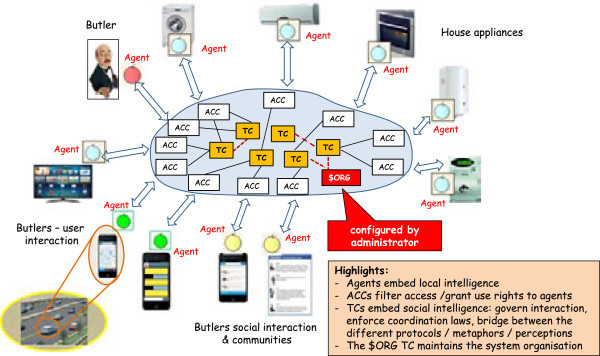
**A possible realisation of the logical architecture shown in Figure** 8 **as a TuCSoN-based architecture.**

For the sake of concreteness, in the next Subsection we commit to a specific enabling technology – the TuCSoN model and infrastructure (Figure 9) – and instantiate the above logical architecture in the TuCSoN case (Figure 10).

### The TuCSoN coordination model and infrastructure

TuCSoN (Ciancarini et al. 1999) (TuCSoN 2013a) is probably one of the most widely used both in international projects (SemHealthCoord 2011) and in the academia – to support virtual enterprises (Ricci et al. 2002), workflow management engines (Viroli et al. 2007), semantic-oriented and pervasive coordination based on MoK (Molecules of Knowledge, (Mariani and Omicini 2012) (Viroli et al. 2010)), up to nature-inspired coordination metaphors (Omicini 2013). It is an open source technology, yet with a license allowing commercial use. It is Java-based, actively maintained, well documented (TuCSoN 2013b), and includes effective tools for distributed monitoring and debugging.

#### The TuCSoN model and infrastructure in a nutshell

TuCSoN adopts a programmable coordination medium, the *tuple centre*, as the fundamental medium to structure the system organisation and govern interaction. Rooted in the basic idea of *tuple-based coordination* (a survey on this topic can be found in (Omicini 2013)), the TuCSoN model supports the coordination of distributed heterogeneous entities according to the objective coordination approach, in a location-aware fashion.

A TuCSoN system is made of *nodes* spread over the network (Figure 9, bottom), which communicate and cooperate with each other, and of *agents* (roughly speaking, processes) operating on such nodes. The system behaviour is governed by the *coordination laws* embedded in the tuple centres, which are designed so as to enforce the desired system behaviour. These laws are expressed in a declarative interaction programming language, *ReSpecT*, which is Turing-equivalent: so, any computable coordination law can in principle be expressed. The most recent ReSpecT version also supports time-aware specifications, as well as sensors and actuators. Tuple centres are inspectable at any time both by agents and humans (via the Inspector tool): in particular, the embedded laws can be changed at any time – provided that the proper access rights are granted – both statically and dynamically. Tuple centres can also cooperate together (the so-called *linkability* property, that is, the ability of a tuple centre to operate on another tuple centre, in a chain), promoting the conceptual and physical distribution of information and behaviour.

The current TuCSoN version also includes a comprehensive *role-based* security model that requires agents to negotiate their roles and permissions both when entering the community and later, dynamically, on a “by need” basis: so, at any time, an agent interacts with the infrastructure via a sort-of “proxy gateway” called Agent Coordination Context (ACC in Figure 9), which filters the agent operation based on its access rights and roles. Thanks to this approach, designers can model the system policies with the desired granularity – typically, based on the organisational view of the system.

#### SODA: an agent-oriented methodology for TuCSoN

In order to inspire and support the whole development cycle of a TuCSoN-based system, from the early requirement specification down to the implementation, a proper (agent-oriented) development methodology should be adopted. Among the several agent-oriented methodologies available in the literature (for survey, see for instance (Omicini et al. 2004)), like GAIA (Wooldridge et al. 2000), Tropos (Bresciani et al. 2004), TOTA (Mamei et al. 2009) and others, SODA (Omicini 2001, Molesini et al. 2006, Molesini et al. 2009) is particularly suited to work together with TuCSoN, since its abstractions and metaphors can be mapped directly onto TuCSoN artefacts.

Although a full description of the SODA methodology is outside the scope of this paper, its general approach can be summarised graphically as in Figure 9: there, the two fundamental phases (analysis and design) and sub-phases of the SODA process are outlined, along with their outcome in terms of relational tables. In short, SODA conceives an agent system in terms of agents, artefacts and societies, that are derived from the initial requirements and later specialised, detailed and concretised along the way; moreover, a role-based access model (RBAC) for multi-agent systems is also considered (Molesini et al. 2009). As a result, the combined adoption of SODA – for the analysis and development process – and TuCSoN – as the corresponding implementation technology – provides an effective tool for MAS design and development.

### TuCSoN for Butlers

#### Motivations

The main reasons why TuCSoN is adequate to the Butlers scenario can be summarised as follows:

- it provides a complete, hierarchical, multi-domain organisational model
- it comes with a comprehensive role-based security model (requirement #5 in Configuration requirements)
- it inherently supports interoperability of heterogeneous agents (requirement #2)
- its programmable coordination media (tuple centres) provide Turing-equivalent reasoning abilities (requirements #6, #7)
- tuple centres also provides dynamic, user-definable, inspectable policies, with the full inspectability of the communication and coordination state (requirement #8)
- tuple centres are accessible and configurable (requirement #3), both locally and remotely, at the infrastructure level
- neither the model nor the infrastructure embed any hardwired technology or pre-installed (locked) configurations
- it is designed so as to prevent bottlenecks (requirement #1).

There are also some possible drawbacks, of course. The first worth mentioning is that accessibility and configurability at the infrastructure level are currently only within the range of expert users, that are sufficiently familiar with the TuCSoN inner structure and languages: however, as discussed in the Reference architecture, this could be faced by adding an extra architectural layer, aimed at filling the gap towards the non-expert user. Another drawback may concern performance: although TuCSoN has proven to be good enough in the applications where it has been used so far, more stressful situations should be tested. This aspect, however, is not crucial here, because our aim is just to present one of the possible implementations of the Butlers architecture with one of the possible available technologies, for feasibility and concreteness purposes – not to support TuCSoN as “the” suggested technology of choice: in principle, any other technology (or a mix of) with analogous features could be used, based on the designer’s requirements and key choices.

#### A TuCSoN-based Butlers architecture

Exploiting the TuCSoN basic bricks, tuple centres and ACCs, the logical architecture shown in Figure 8 and discussed in Section Logical architecture of a possible Butlers platform can be refined as in Figure 10. With respect to the main key aspects outlined above, the TuCSoN choice:

-  enables both one way and bi-directional communication between the entities and the infrastructure;
-  inherently supports heterogeneous protocols, since tuple centres can be programmed to bridge between different protocols and possibly among different agent perceptions; as a welcome side effect, new protocols can easily be added to an existing system, promoting incremental design, modularity, and the integration of new components into legacy systems;
-  being based on the observable behaviour, intrinsically supports any kind of agents, independently of their technology, internal structure, etc.;
-  results in a platform that is inherently distributed yet location-aware, thus providing designers with the power to balance distribution (that is, how many tuple centres should be put in which nodes), and hereby performance, with locality awareness;
-  being based on objective coordination, allows coordination laws to be enforced by the infrastructure, not just depend on well-behaving agents;
-  gives rise to a platform that is completely configurable, where policies are expressed declaratively as ReSpecT programs, are not hardwired once and for all, are dynamically malleable and inspectable, and can be defined both statically and dynamically, both by any human or software agent with the proper access rights;
-  allows users to change/integrate policies at different levels and by different means: while the developer and the expert users can operate directly in the ReSpecT language, end users can potentially be equipped with a higher-level interface providing a more abstract view over the policies, with adequate metaphors to express and manipulate them. Moreover, these views can coexist, both conceptually and practically, and other views can be added at a later time: so, for instance, a prototype system can have initially no higher-level policy manipulation features – the only way to manage policies being the direct tuple centre programming – but be extended later with some “add on” component that makes the higher level manipulation possible.
-  more generally, allows the system to be extended at any time with little or no impact on the existing system, as long as the addition of new components, interaction protocols, agents, and coordination laws is “made transparent” by some suitably programmed tuple centre;
-  inherently supports intelligence in the infrastructure: because ReSpecT is Turing-equivalent, potentially any (interaction-related) computation can be embedded, and any computable coordination law can be computed; at the same time, the chance to spread such intelligence among the system – in particular, to decide what to put onto agents and what onto the infrastructure – where and when needed provides the designer with the full power of tailoring the system according to his/her goals and needs;
-  makes it easy to bridge the gap caused by any source of heterogeneity (different protocols, different languages, different expression levels, etc.), thus also supporting the addition/replacement of entities to the system at later times;
-  supports potentially any kind of social interaction, since the designer can both bridge the gap (as in point *j* above) between virtually any perception, protocol and entity, as well as decide how to split the required logic among agents and the infrastructure (as in point *i* above);
-  from the security and organisational viewpoints, natively supports a role-based access control model that can be tailored to the specific application needs;
-  last but not least, provides an infrastructural item (the ACC) that is specifically thought to define a clear (but negotiable) external boundary, so as to protect both the infrastructure and the system built on its top from the external world.

Because of these features, the specific technologies for implementing the physical communication (e.g. ZigBee rather than Wi-Fi or Ethernet on PowerLine, or wired Ethernet, etc.), for enabling communication at the logical level (communication language, higher-level protocols, etc.), for retrieving measures and issuing commands to remote-controllable appliances, for showing and manipulating information onto a variety of devices (TV screens, smartphones, tablets, up to possibly 3D-enabled devices, etc.), and so on, need not be specified at this level: many of them could be used, possibly also together – it all depends on the vendor’s preferences and goals: the key aspect is that they do not influence the system design, since the infrastructure provides the technology to manage heterogeneity, filling the gaps. As a result, the architecture at this level still accounts for a wide set of systems, yet maintaining the generality of the reference architecture depicted in Figure 1: in principle, different “editions” of the same application can also be imagined, with a different set of supported features – it is all up to the vendor’s goals.

So, the Butlers logical architecture in Figure 8 takes now the form of a possibly-distributed set of tuple centres and ACCs (Figure 10): any external appliance is associated to an agent, connected to an ACC that defines its admissible operations and roles in the TuCSoN system. Other ACCs capture internal agents’ interaction contracts. A special tuple centre, called *$ORG,* contains the specification of the organisation logical structure. Tuple centres embed the coordination laws required to handle the system, enforcing the desired properties and hereby implementing the “social intelligence”: this is further supported by the so-called linkability property of tuple centres, that is their ability to trigger reactions in other tuple centres, according to the local programming rules, as a consequence of a local event. Agents, on the other hand, embed the individual intelligence (if any, when and if opportune) of external entities.

### The sub-system prototype

In this section we focus on a selected sub-system, that is small enough to be implemented as a prototype requiring neither a large team effort nor vendors’ involvement, yet retaining the key aspects of the full system.

#### Requirements and architecture

With respect to the full system, the following constraints and limitations apply:

- one single house is considered, with limited goals – referred only to lighting, air conditioning and heating;
- there is no intelligent Butler recognisable “as such”, like Archie in the comic strips above;
- user localisation is limited to the interior, and is simulated via GUI – that is, no real geo-localisation is actually present;
- social interaction facilities are not supported;
- gamification is not supported, either.

**Figure 11:**
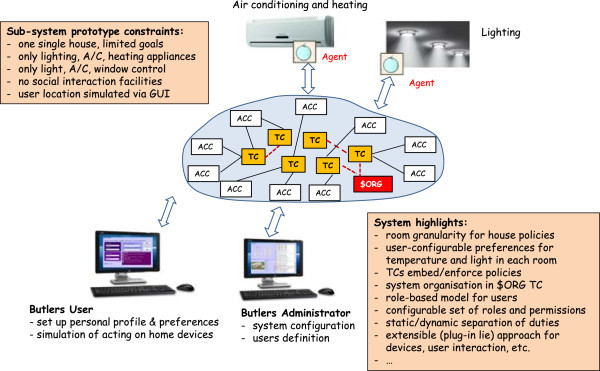
**The small sub-system of the TuCSoN-based architecture shown in Figure** 10 **selected for the prototype.**

The consequent architecture is synthesized in Figure 11. Both the standard Butlers user’s and the system administrator’s interaction are simulated via a traditional computer GUI: users can exploit the GUI for both configuration/personalisation purposes and to simulate actions on house appliances/devices, while the administrator is only concerned with the overall system configuration – in particular, users’ definition. Despite these limitations, the system maintains the desired key features, namely:

- user-defined maximum energy consumption threshold, to be monitored and enforced by the system;
- enforceable coordination laws that govern the interactions and enforce policies among all the involved entities;
- an extensible architecture based on loose coupling, where new devices, entities, and interaction protocols can be added seamlessly and incrementally:
- both comfort-oriented and energy management policies;
- both local (room-specific) and global (house) policies, applied to selected areas (lighting, air conditioning and heating), with support for custom user preferences;
- a full-fledged role-based user model, with a configurable set of roles, and both static and dynamic separation of duties (SSD/DSD constraints).

#### Application scenario

**Figure 12:**
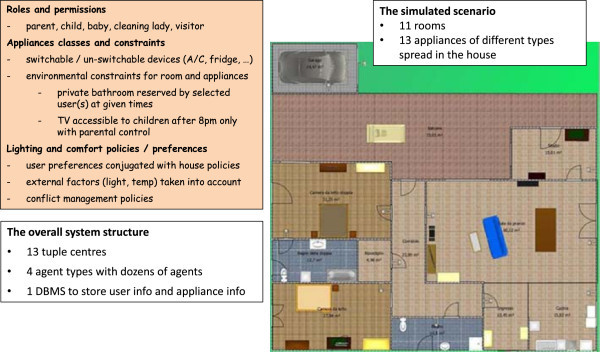
**The intended application scenario of the small sub-system prototype shown in Figure** 11 **.**

Figure 12 presents the intended application scenario, the house plan and the supported user roles, permissions, appliances and policies. The house has 11 rooms (entrance, corridor, kitchen, living room, office, storage closet, bathroom, private bathroom, garage, single bedroom, double bedroom) and a balcony. Rooms are furnished with 13 appliances of 9 different types (home entertainment, heating/air conditioning, freezing, cleaning, cooking, cutting, lighting, infix appliances, maintenance items): in particular, we distinguish switchable devices (like T.V. set, air conditioning, etc.) from un-switchable devices (like the fridge).

There are 6 user roles: *parent* (administrator), *child*, *baby*, *cleaning lady*, *visitor* and *unknown*. Parents have no restrictions – they can access all rooms and use all appliances; as administrators, they also define the house maximum energy consumption threshold, later enforced by the house management system. Children are not administrators, but apart from that have no fundamental restrictions – their only real limitation is the prohibition to access the private bathroom. Babies, instead, are forbidden to access the storage closet and the garage, too, and cannot use cooking or cutting appliances: moreover, they can access home entertainment appliances only during the weekends, until 8 pm, and with parental control. The cleaning lady can access all rooms and use all appliances, but only 8:30 am-6:30 pm Mon-Fri. Visitors have the same basic rights as babies, but can use appliances only in the presence of an adult living in the house (i.e., parent or child): in this case, however, special exceptions may be needed – for instance, if the visitor is a repair man or a technician who needs to access a normally-forbidden room or use a normally-forbidden appliance to perform his work: it is then up to the administrator to define specific, ad hoc policies. Finally, the unknown role represents unrecognised people: since these should not be in the house, their presence is immediately reported to parents and children (in principle, via mail and/or SMS, etc.).

Environmental constraints apply to both rooms and appliances: for instance, the private bathroom is reserved to specific users at given times, but is available to others when the intended users are not at home; the TV is accessible to children in the evening and night (e.g. after 8 pm) only with parental control; and so on.

User preferences are taken into account whenever possible, but only as far as the general house policies and the room policies allow. These policies, in their turn, may take external environment factors, such as light and temperature, into account. Proper meta-policies are also defined to mediate among possibly conflicting policies. For instance, some policies prescribe that:

- the room lights are switched on only if the room light sensor acknowledges;
- the room temperature is set at “approximately” the average of the temperatures desired by all the people currently in the house, where “approximately” means that the heating/air conditioning are switched on only if the desired temperature differs from the actual one for more than 3°C;
- in case of conflicts, the user that has been in the room for the longest time prevails.

Every time a user enters a room, his/her preferences are taken into account, and the room condition is re-checked trying to meet the user’s expectations; of course, unknown people are not pandered to – room lights remain off, etc.

#### Implementation as a TuCSoN multi-agent system

**Figure 13:**
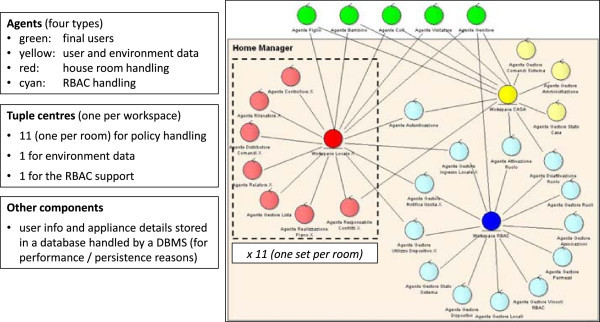
**The architecture of the small sub-system prototype as a multi-agent system.**

To implement such a system as a TuCSoN multi-agent system, the SODA analysis and design process has been applied twice: first, to the RBAC engine in general; then, to the house management system in particular. While its details are outside the scope of this paper, the resulting MAS is shown in Figure 13: thirteen tuple centres – one per room, plus one for the RBAC engine and one for environment data – are in place, together with several dozens of agents, conceptually grouped into four main categories drawn in different colours: final users (green), data handling (yellow), room handling (red), and RBAC handling (blue/cyan).

For the sake of concreteness, we report below the specification of an ACC contract – one of the many around – namely, the one belonging to the *Role Activation Agent* (one of the cyan agents in Figure 13) which checks the preconditions for role activation. Apart from the details, it is worth noting the set of can_do rules, that express what the agent is allowed to do, possibly specifying under which conditions, in a declarative way.

This code checks 1) that the role is not active for the same user, 2) that it is associated to the requesting user, and 3) that the DSD constraints are respected.

Analogously, the code below shows a small excerpt of tuple centre programming – a subset of the ReSpecT reactions of the RBAC_TC tuple centre (the blue one in Figure 13) that takes care of deleting all the information related to a role to be cancelled.

Both code chunks refer to our current implementation on TuCSoN 1.4, which still adopts the ReSpecT 1.5 syntax: a refactoring to move the implementation onto the novel, better performing TuCSoN 1.10, embedding the extended ReSpect 2.x syntax, is underway.

In addition, both to stress-test the support for heterogeneity and the interaction with a legacy system, the plethora of detailed user data and the appliances’ details are stored in an external conventional data-base management system, not internally to the TuCSoN infrastructure.

**Figure 14:**
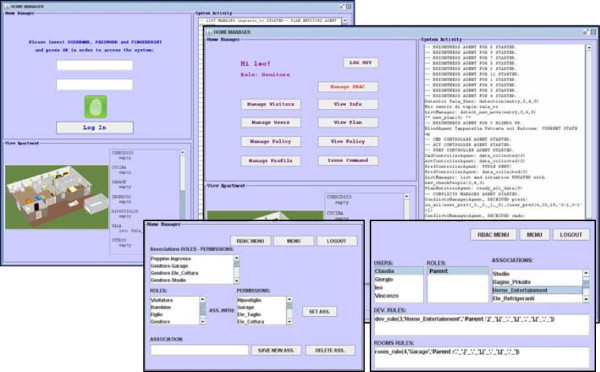
**Some screenshots from the implemented small sub-system prototype.**

Figure 14 shows some screenshot from the implemented prototype – the login dialog (top left), the user dialog (top right), and the configuration dialogs for the roles handling (bottom). Both the top windows include a system activity log on their right bar, for monitoring and debugging purposes. This user (in this case, *Leo,* a parent, therefore with administrator privileges) can view various kinds of information, manage users, roles and policies, and issue a command to simulate an action. The bottom dialogs enable the management of roles/permissions associations (left) and of roles/users associations (right): the latter also shows the corresponding rule expressed in the Prolog-like formalisation adopted.

### Discussion and up-scaling roadmap

Admittedly, the prototype presented in this section is a small subset of a whole Butlers system, with considerable limitations: so, it is worth discussing how far it is – both conceptually and practically – from a full Butlers system.

#### Appliances

Appliances in the prototype are totally simulated – we currently have no physical interface to any real appliance. Yet, the technology survey in Section Technologies showed that several technical solutions to monitor and remotely control appliances of any kind are available on the market. Therefore, integrating real appliances in the system is feasible – whatever communication protocol and interaction language they use – since the TuCSoN infrastructure fully supports heterogeneity from these viewpoints and can bridge the possible gaps. The required effort amounts to writing the proper “driver” software in terms of a) suitable tuple centre and ACC programming, and b) the appliance interface agent, whatever its implementation language and running platform are.

#### Users

Users, and user movement inside the house, are also simulated – there are no software interfaces to GPS and other localisation systems. Yet, geo-localisation technologies are embedded in smartphones, and other low-cost technologies such as movement sensors, touch sensors, fingertips sensors, etc. are available and could be used inside a building. External positions could also be grabbed from social networks like Foursquare, Facebook, Twitter, etc., provided that the necessary software is set up.

So, while the integration requires the development of an adequate software layer in the form of a suitable proxy agent and corresponding TuCSoN programming, as above, it is clearly within reach. Since the burden of properly handling the heterogeneity of such sources can be left to the infrastructure, multiple different technologies could be used, even simultaneously, and integrated: suitably-programmed tuple centres could also provide a unified view of a user’s positions independently of the specific underlying technology exploited to get the location information at a given time – thus integrating information coming from GPS or Wi-Fi (inside and outside the house), house sensors (inside the house), social networks (mainly outside the house), etc.

#### Policies

Policies in the prototype are currently limited to a few pre-defined fields (heating, air conditioning, lighting), but others could be added by writing once again the necessary specifications in tuple centres/ACCs.

Since policies are more likely to change at run time with respect to the appliance types or user localisation technologies above, which are more stable by nature, an administrator – or someone else with proper access rights – should be enabled to operate on tuple centres and ACCs, either directly or indirectly, also at run-time, so as to tune and configure the system dynamically. (Of course, once available, this feature could in principle be exploited to introduce further appliances and user technologies, too). Moreover, if selected end users, other than administrator, are to be enabled to add/change (some kinds of) policies, a higher-level user interface, with adequate metaphors, has to be designed to support them in this task, as it would be unrealistic, and dangerous, to let them operate directly on the infrastructure at the basic programming level.

In the current basic prototype, the access to tuple centres and ACCs is generally done at the infrastructure level, and is therefore only within the reach of an expert user; at the same time, for the above reasons, an effort has been made to make a step towards a more user-friendly approach, as demonstrated by the dialog windows shown in Figure 14 (bottom) for role handling. In fact, although such dialogs are domain-specific and still require a clear understanding of what a role is and of the purpose of role/permission and role/users associations, therefore being aimed at skilled users, they do enable parents to define role-based policies without writing low-level (i.e., ReSpecT) code themselves. Of course, such a feature is unnecessary for closed systems, that are by definition configured by the vendor/installer and then simply “used as they are” by the end users. Since the Butlers architecture allows both kinds of systems, whether such a feature is necessary or not depends, once again, on the vendor’s goals and market targets.

#### User-side configurability

User-side configurability requires both proper metaphors and a suitable software support, aimed at making the configuration process understandable for various categories of end users, that are not – and should not need to be – computer experts. A variety of interaction systems could be considered, enabling configuration from different physical devices at different abstraction levels, possibly with a different level of detail on different devices and/or for different user categories: as an example, computer screens and smartphones could offer a complete UI with plenty of details that is mostly suited to expert users, while 3D TV screens and gaming consoles with movement detection could provide a very different, yet involving, user experience that turns the configuration process into something “physical” and “touchable” – a sort-of 3D game where items representing configuration aspects can be moved and composed in some kind of virtual world for non-expert users. Proper abstraction levels would be needed based on the user’s age, culture, etc.: for instance, a child wishing to configure the lighting or the music/media player in his/her room could be provided with metaphors like pets, toys, puppets, games, and other familiar objects from his/her everyday life, while his/her grandma’s and parents would likely need rather different abstractions; and so on.

Although the current prototype does not include any of these features, the mediated interaction approach makes their addition an orthogonal aspect: as long as the proper specifications are eventually written in the proper tuple centres, the infrastructure itself does not care of how and where the specification themselves originated – be they written directly as ReSpecT code by an expert, or synthesized by a GUI like the ones in Figure 14, by some advanced 3D-UI in a gaming console, etc. That said, the design and implementation of such a support, that involves most of the Butlers layers, up to gamification – can be well expected to demand much effort both conceptually and industrially: the road to its realisation will be neither short nor simple. Yet, the required technologies are already in the users’ homes, so in principle they “just” need an enabling technology to glue them together from the outside, masking and bridging their differences – precisely what TuCSoN is for. In this context, a vendor could develop a “Butlers module” aimed at supporting the configuration of, say, child rooms based on the pet metaphor, for, say, Xbox Kinect™ or other similar platforms, and distribute it together with the corresponding TuCSoN interface software – or anything else: the key requirement is a clear idea of the target users, on the one side, and of the value-added provided by each technology, on the other – namely what the Butlers reference architecture (Figure 1) means to provide.

#### Butler

The last crucial item which is totally missing from the current prototype is the (possibly humanized) Butler, like Archie and John in the comic strips. As long discussed above, this component is supposed to embed the higher-level intelligence, anticipating the user needs, possibly learning as time goes by, and possibly interacting with other butlers various ways to exchange experiences, best practices, etc.

Conceptually, the prerequisite for these tasks is that the whole state of the system and of the interaction is reified, inspectable, and available to the Butler at any time, so that any kind of deduction can in principle be supported: TuCSoN satisfies this constraint, since all the relevant information is stored in tuple centres. Moreover, tuple centres can also be programmed so as to highlight the events, facts, and issues that are specifically relevant for the tasks to be performed, helping to build ad-hoc views that are tailored to the desired abstraction level. On the other side, the butler itself can be seen as a sort-of autonomous, intelligent agent embedding specific skills in selected areas (e.g., energy consumption optimisation, etc.): if the tasks to support is particularly complex, in principle it could also take the form of a society of cooperating agents. Technically, any AI model and system from the many available in the literature and AI research could be adopted: thanks to the mediated interaction approach, this is once more an orthogonal choice.

Depending on the vendor’s target and marketing policies, butlers with increasing competence and abilities could be developed that include a different set of Butlers layers (as discussed in Section Notable scenarios and system patterns), supporting different “editions” of a Butlers systems. Proper standards could also be established for the butler’s interaction, so that butlers from different vendors may interchange and inter-operate. While this perspective is outside the scope of the Butlers architecture *per se*, it could constitute another dimension worth investigating from the industrial viewpoint.

## Conclusions

In this paper we put together house energy management with domotics, intelligent agents, ambient intelligence, ubiquitous technologies and gamification to depict novel scenarios where today’s pervasive technologies provide an innovative, comprehensive user experience enabling intriguing, advanced features. In the gamification perspective, we put special emphasis on the entertaining aspect and the social involving of new technologies to improve their acceptation and diffusion.

As widely discussed above, there is an increasing industrial interest both on energy monitoring, controlling and saving systems, on the one hand, and on their integration with intelligent appliances (LG Newsroom 2013), smart homes and smarter lifestyle, pervasive/ubiquitous services, on the other – see for instance the “personal concierge” scenarios in (NewsPanasonic.Net 2013). Most leading vendors are developing their solutions, systems, projects, approaches to face these needs. In such a dynamically moving context, the main contribution of the Butlers architecture is probably its united and systematized view over a wide variety of possible systems and application areas, including aspects – like gamification – that are currently unconsidered by industrial systems, but that can be well expected to find their role in the foreseeable future.

Another Butlers’ key feature is the independence of its multi-layer reference architecture from any specific technology, which relates the value-added for users to the expected features and to the technical requirements in a technology-neutral way. This is especially relevant in today’s continuously-evolving context, where new technologies and products appear every few months, with the related heterogeneity and legacy issues. Moreover, the Butlers layers may act as a sort-of indirect industrial comparator and suggester tool, in three main ways:

1. to clarify the “conceptual location” of a given system in the hierarchy, based on the set of features it provides;
2. to compare the features and value-added provided by different systems from different vendors, possibly suggesting extensions and third-party add-ons;
3. conversely, to highlight possible “free slots” in the systems hierarchy – that is, “possibly missing” industrial products, thus highlighting new opportunities worth investigating for new systems aimed at supporting uncovered features.

At the same time, this is admittedly a visionary work: while potentially we are not that far from the Butlers scenario, because all the necessary technological ingredients are available today – including the coordination technologies to “glue” so many different and heterogeneous systems together – , the road towards the realisation of a complete Butlers system will not be straightforward. First, the effectiveness of the 7-layer architecture needs to be proved on the field, both conceptually and practically, beyond the boundaries of the prototype implementation example discussed here. Moreover, several non-trivial issues need to be dealt with, both on the conceptual side (in particular, how to represent abstract concepts as 2D/3D entities in the configuration and specification processes, how to integrate the reasoning layer with a suitable coordination layer, just to cite some) and on the practical side (e.g. how to properly integrate heterogeneous systems and technologies). Performance and robustness, in particular, will be key success factors. Of course, such aspects cannot be reliably evaluated on the current small prototype, not only because of its limited size and goals, but especially because the Butlers architecture accounts for many possible different systems, each with its own policies and objectives: so, vendors could make very different design and technical choices, with very different trade-offs between cost, performance, reliability, robustness, etc.

It could also be worth highlighting that the Butlers architecture is not primarily aimed (only) at energy saving: it takes a much wider perspective – the comprehensive home management – where many other aspects are considered and integrated. So, while energy saving can be expected to be one of the most typical policies, a Butlers systems could well opt for (i.e., be designed and configured for) other goals and priorities – for instance, the maximum user’s comfort – which might potentially lead even to the opposite result – higher energy consumption at some times. These choices do not depend on the Butlers architecture: since the Butlers layers account for many possible different systems, each specific system reflects its vendor’s objectives, as well as the user’s preferences and desires. As a result, whether a given Butlers system leads to a gain in the environment, or helps in the sustainable living perspective, cannot be said in general: the potential is there, but its reification depends on the specific policies. The intriguing point, however, is that the widespread intelligence available in the most complete scenarios – namely, the Intelligent Butler, the Social Butler, and the Comprehensive Butler –, together with the knowledge of the user’s habits and his/her current position, allows for many potential optimisations in several directions, beyond the “pure” home management. For instance, the Butler could provide suggestions to the user in his/her everyday life, such as a home/work commute path that takes into account an extra intermediate stops at the supermarket (because the fridge signalled such a need) so as to save a further car trip, etc., thus promoting virtuous behaviour and overall contributing to a better living; and so on.

For all these reasons, we believe that the perspectives discussed in this paper, despite the clear above-mentioned limits, are in the nature of things: technology is becoming more and more pervasive, awareness and intelligence in energy consumption, and more generally attention to the environment, are first-page issues everywhere. Our desire and expectation to control everything easily, remotely and from any kind of portable device at any time is also increasing, and so are our social presence online and our willingness to share where and with whom we are, and be possibly tracked for such purposes. So, eventually these aspects can be expected to merge – and home management, in all its many aspects and nuances, is too a killer application to miss it.

## Endnotes

^a^In the USA and Canada, customers’ groups exist that claim possible health problems due to wireless interference from smart meters, claiming the right to switch back to analog meters. The debate is going on – see for instance (Mercury 2012) (Global Research 2012) (Stop Smart Meters USA 2012) (Stop Smart Meters Canada 2012).

^b^For instance, in Italy the standard home contract is 3 kW, although higher, more expensive offers are available; in France contracts are available for 3-6-9-12-15 kW, with minimal price differences between the lower sizes. Of course, higher thresholds are usually chosen by users who use electricity for several appliances – e.g. electric cooking instead of gas-powered cooking, electric heat pumps instead of other gas-based or oil-based heating, etc.

^c^The word was first introduced by Jesse Schell at the *D.I.C.E. (Design, Innovate, Communicate, Entertain)* Conference, in Las Vegas (NV), 2010. See (Gamification 2012)(Schell 2010) (Schell 2011) for further references.

^d^This approach is used for instance in real-time bus information systems, like the *HelloBus* service in Bologna (Italy): the user sends a message with the bus stop number (e.g. 44) and line number (e.g. bus route 32) in a form like “44 32”, possibly adding the desired time (e.g. “44 32 1100” for 11 am), and gets the arrival time of the next two buses in response.
